# The meaning of living close to a person with Alzheimer disease

**DOI:** 10.1007/s11019-016-9696-3

**Published:** 2016-03-18

**Authors:** Mette Bergman, Caroline Graff, Maria Eriksdotter, Kerstin S. Fugl-Meyer, Marja Schuster

**Affiliations:** 1 Department of Neurobiology, Care Sciences and Society, Center for Alzheimer Research, Division of Neurogeriatrics, Karolinska Institutet, 141 57 Huddinge, Sweden; 2 Department of Neurobiology, Care Sciences and Society, Center for Alzheimer Research, Division of Clinical Geriatrics, Karolinska Institutet, 141 57 Huddinge, Sweden; 3 Department of Geriatric Medicine, Memory Clinic M51, Karolinska University Hospital, 141 86 Huddinge, Sweden; 4 Department of Neurobiology, Care Science and Society, Division of Social Work, Karolinska Institutet, 141 83 Huddinge, Sweden; 5 Division of Social Work, Karolinska University hospital, 141 86 Huddinge, Stockholm, Sweden; 6 Department of Technology & Welfare, The Red Cross University College, Box 55676, 102 15 Stockholm, Sweden

**Keywords:** Alzheimer disease, Early-onset, Interpretative phenomenology, Lifeworld, Spouses, Professional approach

## Abstract

Only a few studies explore the lifeworld of the spouses of persons affected by early-onset Alzheimer disease (AD). The aim of this study is to explore the lifeworld of spouses when their partners are diagnosed with AD, focusing on spouses’ lived experience. The study employs an interpretative phenomenological framework. Ten in-depth interviews are performed. The results show that spouses’ lifeworld changes with the diagnosis. They experience an imprisoned existence in which added obligations, fear, and worry keep them trapped at home, both physically and mentally. In their longing for freedom, new strategies and attitudes helps the spouses to create an extended “lived space” with their partner. The findings stress the importance of paying attention to the lifeworld of spouses and making clinical recommendations on this basis. Most importantly, the lifeworld perspective has implications for how we understand what care is. We hope to challenge all different healthcare professionals and invite them to discuss the deep meaning of care and the definition of being professional in encounters with vulnerable others from a lifeworld perspective.

## Introduction and previous research

On my way home I see all these happy people, walking hand in hand, laughing and smiling. I can’t stand seeing other people being happy together, if they only knew how everything can change …

Early-onset dementia affects people younger than 65 years and little is known about conditions for these early-onset dementia families (Ducharme et al. 2013). Studies often focus on elderly people, older than 65 years, and most research has focused on spouses as caregivers and their care burden. This study has a more holistic, lifeworld perspective with focus on experiences of spouses to persons younger than 65 years suffering from Alzheimer disease (AD).

When someone suffers from AD the entire family is affected (Parahoo et al. 2002). AD is a progressive disease that affects the brain and the most common symptom is memory loss. Other functions that can become noticeably reduced are the understanding of written or spoken language, the ability to express oneself, spatial orientation, and practical skills (Thies and Bleiler 2013).

Systematic reviews show that the problems arising from the development of AD are especially difficult for early-onset families (SBU 2008). Compared to spouses of older people with AD, spouses of early-onset AD individuals may have responsibility for the family, finances, elderly parents, and young children as well as being working professionals (Lockeridge and Simpson 2013). The symptoms associated with the development of AD may be the same in both groups, but the emotional reactions and frustrations are different for families of early-onset AD patients compared to those of elderly AD sufferers (Ducharme et al. 2013).

It is important that spouses receive emotional support to reduce their depression, anxiety, guilt, and other negative psychological symptoms and to help them with psychosocial and financial consequences (SBU 2008). Husbands or wives that still work and also support and care for a loved one with AD can experience emotional and physical exhaustion (Luscombe et al. 1998). Spouses of individuals with AD tend to decline support. However, they also complain that they lack someone to talk to about their situation, and express a wish to meet others with similar experiences and health professionals who understand their situation and have the time to listen (Lockeridge and Simpson 2013). The more difficult their loved one’s symptoms are, the greater the risk that relatives will suffer from depression (Mausbach et al. 2006). Sometimes the feelings of shame they experience about their situation may prevent them asking for help. When they finally do, their partner’s symptoms may have become so severe that spouses need significant amounts of help and support and are completely exhausted (Parahoo et al. 2002).

An AD diagnosis may affect the marital relationship and spouses can find it difficult to achieve a balance between being a partner and being a caregiver. In addition to support and care for their loved one, spouses experience grief and fear of losing their partner, close friend, and lover (SBU 2008). Previous studies show that there is a lack of both psychosocial and formal support for spouses (Gaugler et al. 2004). Spouses who have trusting, close, and regular contact with a support person suffer less from depression than those who lack this support (Waite et al. 2004).

To conclude, earlier studies have described the life situation of spouses and other relatives as mentally, physically, and financially burdensome. Feelings of guilt, loneliness, and powerlessness are difficult to bear. Only a small number of studies have sought to understand the life of spouses from a holistic, lifeworld perspective. Adopting this perspective but with focus on elderly couples, findings showed that spouses felt a change from togetherness to loneliness and that both persons with dementia and their spouses perceive themselves as strangers in their own world. They no longer have an obvious existence and they feel themselves bewildered in their life situation (Meyer et al. 2016; Svanstrom and Dahlberg 2004). In another study, with the same focus on early- onset dementia as our study, the findings showed that the spouses had difficulties to manage different symptoms connected to the diagnosis. Long quest for diagnosis, nondisclosure to others and denial of diagnosis were other findings. The spouses experienced grief, had problems with the unexpected role as a spouse and daily life responsibilities, and difficulty to plan for the future (Ducharme et al. 2013). Other interesting studies with more holistic perspective, but with focus on persons suffering from dementia, investigated family care-givers’ efforts to preserve the personhood of individuals with advanced dementia when they were moved to a long- term care facility (Palmer 2013) and the experiences of younger persons who lived at home and suffered from early-onset dementia (Rostad et al. 2013).

It is important to understand the different needs of the spouses to plan either individual or group interventions and to determine if there is a need for medication or other treatments (Mittelman 2003; Yin et al. 2002). However, a lifeworld perspective as a point of departure to understand the phenomenon of being a spouse to a partner with early-onset Alzheimer raises issues beyond interventions, medication and treatments, namely issues concerning our human vulnerability. It actualizes questions about how healthcare professionals understand and deal with their obligation to alleviate suffering. To have a lifeworld approach to caring is especially important in care settings where questions of meaning and meaninglessness are constantly present. Therefore, more studies are needed to gain knowledge and understanding of spouses’ life situation from a lifeworld perspective.

### The aim of the study

The aim of this study is to explore the lifeworld of spouses when their partners are diagnosed with early-onset AD. What does it mean to live with a partner suffering from AD?

### The phenomenological framework

On the basis of our research question with focus on how people make meaning of their lives, a qualitative approach is suitable. Philosophical theories of phenomenology are applied to describe and understand the meaning of lived experiences of the spouses.

We are exploring the spouses’ lived experiences in their everyday world, their lifeworld, a concept introduced by Husserl (Dowling 2007). Heidegger developed Husserl’s theory of the lifeworld and our inevitable involvement in it (Ashworth 2006). He describes the lifeworld as a subjective perspective, a social reality in which people relate and interact (Dowling 2007). Heidegger illustrates this impossibility of being disconnected by his concept of being-in-the-world (McConnell-Henry et al. 2009). The idea of lifeworld is complex and abstract. To explore it we need to delineate and define it more.

Several phenomenologists have thematized interrelated elements of lifeworld in different ways (Ashworth 2006). Van Manen (1997) describes, with reference to Heidegger, four common themes that can be seen as the existential basis of how all human beings perceive and exist in their world. They can be understood as the fundamental structure of the lifeworld.

The “lived space” relates to spatiality and refers to the perceived surroundings and our place in the world. Our space experience affects our feelings and being. The “lived body” is a representation of ourselves by which we can gain access to the world. Through the body, we experience the world and it is through the body that we encounter others. The body can separate us from or unite us with others.

The “lived time” refers to subjectively perceived time rather than objective time, which can be measured and calculated. Lived time is a person’s interpretations of the time dimension; the past, present and future containing memories, future hopes influenced by the present. This constitutes the temporal landscape. Finally, the “lived human relationship” is formed by how we experience others in deep relation to ourselves. Together with others, we seek security and meaning in everyday life. The experience of the lived human relationship is personal mirroring ourselves and the other (van Manen 1997).

The lifeworld perspective focuses on our living conditions in the present and how we experience and describe them (van Manen 1997). Svenaeus (1999) describes how the existential depth of our lifeworld becomes apparent in situations marked by suffering, guilt, struggle, and death. Death can be seen as a prerequisite for understanding life itself; becoming aware of the reality of our mortality might create anxiety, a feeling of homelessness in existence. Disease and the threat of death affect our being-in—the world and suffering pushes us out of our comfort zone. The experience of homelessness—our fear of death—is an existential aspect of life. We are all destined to experience this predetermined homelessness that is beyond our control.

## Method and design

### Interpretative phenomenology

To explore the lifeworld means to examine the immeasurable. This study has an orientation towards creating meaning by achieving a deeper, more holistic understanding of the phenomenon of being a spouse to a person with early-onset AD. This means that we must move beyond descriptions of lived experiences of the spouses and interpret them, to “read between the lines” (Finlay 2009) without losing sight of what stands on the lines. Therefore, we turned to interpretative phenomenological reflection, a method introduced by van Manen. This is a process with focus on reflection, clarification and structure, using interviews and reflective interpretative analysis to gain a deeper understanding of the spouses’ lifeworld. With interpretative phenomenology we understand the being of a subject as always existing in the world, anchored in time, place, social relations and body. Instead of looking for an essential structure of the meaning of living close to a person with Alzheimer disease we strive for understanding different ways people experience the phenomenon.

Lifeworld existentials as described by van Manen were important tools guiding us throughout the reflective research process, from interviews to analysis and interpretation of the data. They offered “a lens through which to explore lived experience” (Rich et al. 2013). The existentials mediated between theory and method, an area that otherwise can be filled with insecurity, confusion and contradictions (Finlay 1999). This means that they helped us to balance between the open attitude so important in phenomenological research and at the same time maintaining a focus on the phenomenon explored. Using the existentials when emerging from description to interpretation of the lived experiences also enabled us to some extent control our pre-understanding. They supported us in the reflective movement between nearness and critical distance to our data by helping us “stepping back from the data and considering the wider and more subtle aspects of the lived experiences” we were exploring (Rich et al. 2013).

### Participants

The participants were recruited through our network of professionals working in dementia care in Sweden. We informed potential participants about the study’s purpose and an information letter was handed out to those who expressed an interest in participating. AD is a progressive disease but it develops individually over time. Criteria for inclusion were participants of working age (aged 40–64 years), partners of and living with persons with diagnosed AD one year or more after diagnosis. The last criteria was important to ensure that the participants had the lived experience and that the diagnosis was not newly given. Consenting participants contacted us and we agreed on a time and place for the interview. Ten spouses were interviewed, five women and five men. All the spouses were working, caring also for elderly parents and six of the participants had children in the age of 18 years or more not living in the household. Two of the participants had grandchildren.

### Data collection

The first author conducted all the interviews, which lasted between 60 and 95 min, with short pauses. A tape-recorder was used and the interviews were conducted individually in an environment chosen by the participant. The interviewer used a conversational method described by van Manen (1997). The method helps both the interviewer and the interviewee to stay close to the research question and the lived experience by focusing on situations, persons and events involved in their experience. Therefore, all the interviews contained the same initial question and just a few probing questions. The first question was “How did you meet?” The interview then continued to focus on the present, on what it was like to be a spouse in a relationship with a partner diagnosed with AD. The probing questions were “How did you feel? Can you describe a concrete situation?” The four lifeworld existentials—the lived body, the lived time, the lived space, and the lived human relationship featured as an internal reflection guide (van Manen 1997). Being observant to these themes in spouses’ descriptions and asking open-ended questions related to them helped the participants to maintain focus on the lived experience of their life situation. This open and exploratory attitude is essential to provide space and time for the participants to go deeper into their experience (Mackey 2005).

### Analysis

The interviews were transcribed verbatim and aspects of non-verbal communication, such as pauses and sighs, were documented to maintain the authenticity of the spoken words in the written account. This is valuable in reflective analysis. It helped us to remain in compassionate contact with the spouses’ experiences as they were transferred into text. The texts were read and re-read by the first and the last author and possible interpretations were discussed throughout the whole process of analysis. The role of our pre-understanding was acknowledged and questioned as we construct the world from our earlier experiences and background (McConnell-Henry et al. 2009). Pre-understanding relates to how our understanding of the world is based on our subjective interpretations, which in turn must be seen in a social and historical context (Mackey 2005). The reflection of one’s own subjectivity becomes a tool in the research process (van Manen 1997) when description meets interpretation. In the Heideggerian tradition, this is both desirable and unavoidable in the development of new knowledge and understanding (Lowes and Prowse 2001). The four existentials mediated between theory and method in the interpretative analysis making a deeper understanding of the spouses’ everyday life possible (Lowes and Prowse 2001).

Themes were formulated by the first and the last author manually, through a selective reading approach (van Manen 1997) to uncover aspects of being a spouse to a partner with AD. All interviews were first reflected on and analyzed individually and we marked statements that described an experience of the phenomena. These statements were then compared with descriptions of similar experiences in order to reveal a pattern. The analysis led to sub-themes with slightly different focus. These sub-themes were then combined into themes that illuminate the complexity of spouses’ descriptions of their experiences. The four existentials provided a framework of the findings, to deepen the interpretation through reflection on how the themes correspond to the structure of the lifeworld.

To conclude, the reflective analysis can be described as a non-linear process, moving back and forth in the data between description an interpretation, experience and abstraction, to explore the researched phenomenon. Throughout the process we asked three questions which “kept us on the road” and supported us in our strive for phenomenological openness. 1. What was it that the text tries to tell us? Here we strived to be near the text with an open and compassionate reflective attitude. 2. How come we understand the text as we do? This more critical self-reflection helped us to question our pre-understandings “separating out what belongs to the researcher rather than the researched” (Finlay 2009). 3. How can we understand the text in relation to our common human conditions of life, the lifeworld existentials? This theoretical and abstract reflection has the ambition to describe and understand the phenomenon at more general level. This means that individual experiences reflected and interpreted against a background of universal human conditions opens for deeper and hopefully new understanding what it means to be human in difficult, life changing situations.

### Findings

The analysis resulted in four main themes: *the experience of being restricted, the experience of being evolved, the experience of being alone* and *the ambiguous experience of being together.* The themes have several sub-themes. Due to the holistic character of lifeworld the themes must be seen as interrelated. The quotes used in the analysis of the different themes could even refer to other themes.

### The experience of being restricted

The sub-themes, heavy responsibility, additional obligations, and a longing for freedom illustrate a state of being restricted. Spouses state how their situation was restricted in various ways by worry, growing responsibilities, and new obligations. They describe how in everyday life this ties them to their home and partner in an intrusive way, both actually and mentally. They have to find ways to endure the present. The spouses long to spend time on their own as a way to break free from their imprisonment.

One of the spouses describes the change in his everyday life:

When I try to go away, if only to the store, I feel worried. I am worried that something might happen at home and I am not there. One time… she wanted to bake and I had to save the burnt cake from the oven…

The spouse describes an experience of worry and heavy responsibility and expressed a fear of losing control over everyday life at home. These worrying thoughts interfere with his life and tie him to the home.

Another spouse describes her situation:

Now everything he does causes disorder and confusion. Among many other things, he loses his keys and glasses … and the bills! He used to take care of our bills and finances but now I first have to find them and then sit beside him and patiently help him to do it right. It takes hours.

When I feel as if I’m drowning … I take a walk.

The spouse experiences her partner as increasingly disorderly and dependent. She needs to take care of extra obligations that are time-consuming and this is demanding and restricting for her. She tries to help her husband in pedagogical ways to manage the situation, to avoid chaos in the present.

The experience of limitations and demands is expressed by one spouse in terms of the desire to be alone:

What if I could get some time with myself? I am ashamed that I feel like this, but it is not as bad as it may sound. I just need to be alone for a little while…

The spouse excuses herself and describes feelings of shame because she longs for something else, a bit of time for herself. Her own world and her social life are limited and she longs for freedom, to be alone to have the time to recover.

### The experience of being evolved

The sub-themes, new connections, changed attitudes, and new strategies are expressions of being evolved.

Challenges in their current life require spouses to change their attitudes and develop new strategies. Encounters with other people who support them are essential to be able to manage everyday life. The spouses create harmony and continuity with past activities by reaching out to others.

The importance of relations with other people outside the home is described by a spouse:

After all that has happened, I am trying to support myself; I need the strength to cope outside… to find my own place. The support group is very important and I would not have made it without them. I prioritize the meetings with the group; it gives enormous strength and people I might never have met otherwise are my best support.

To take care of herself, the spouse finds a context outside the home with new connections. It is important for her to gain strength through her own activities. Social interaction with others who understand her life situation becomes essential.

The spouses are forced to develop new attitudes to be able to cope with their changed life-situation:

I try to minimize the conflicts; there is no point. We want happiness and harmony in our relationship and in our lives. I must remember how important it is to not contradict or correct him when he is wrong.

The practice of minimizing conflicts is crucial for a good relationship. The spouse describes how she prioritizes a happy atmosphere and how she changes her attitude to generate harmony and create good memories.

Another spouse states:

Back then we lived a good life. I was fishing, and she stayed in the cottage or on the beach. Now, my fishing requires a lot more planning; I need, for example, to try to bring someone who can join her so I can relax for a moment on the lake.

Activities that were good in the past have to be realized with new strategies and by the spouse’s own effort and motivation. The spouse expresses a new creative side of himself of which he had previously been unaware. He is now inventive and flexible to retain what once was.

### The experience of being alone

The feared loneliness, the need for others, and the desire for solitude are sub-themes that illustrates a state of being alone. To be alone has a dual nature; it is both devastating and long-awaited. The spouses describe the fear of loneliness, of being lonely and abandoned, but also a desire for solitude, expressed as a need of breaking away mentally from their current life situation.

One spouse describes his feelings of abandonment and loneliness:

At night when she sleeps, I hug her, feeling her scent and I beg, beg her not to leave me. There is nothing I can do, she has in a way already abandoned me… When I think about it I get angry instead, life is so unfair.

The spouse tries to maintain the image of his beloved. He describes an existential anxiety about how he doesn’t want to let go and how he tries to hold on to her at night to get her back. He tries to preserve the image of her as she once was, under the cover of darkness.

The need for others, being with others without her loved one, is expressed by one spouse:

My breathing space is my bridge evenings; once a week we meet and the hours fly by. I’m enjoying every second: my friends, the chatting, and focusing on the game. It provides a mental rest; I relax while I apply myself to the game.

By meeting with friends and playing cards, this spouse can relax mentally. While she is enjoying herself, she can focus her mind on something else and she regains her energy.

One spouse illustrates the importance of being alone in another way, as a desire for solitude:

My jogging keeps me in shape. To be alone with myself, just to run, gives me a wonderful feeling of just being. I clear my brain, thinking of nothing and I just run. It is my way of surviving… I have always found peace in running; it strengthens me mentally and I will cope. I have to be in shape, both physically and mentally.

The spouse describes how he runs for his survival, how he runs for his life. Being alone, in the solitude of running, gives him an inner peace and helps him to endure.

### The ambiguous experience of being together

The sub-themes, loss of intimacy, being close, and feeling a sense of kinship are different expressions of being together, illustrating the complexity of both being together and yet not being together. The spouses describe the need for togetherness with their loved ones that is both difficult and profound. The old ways of relating have changed. They experience a loss of closeness when they helplessly try to reach out to their loved ones. However, a kind of intimacy is still found in a wordless relationship, showing a deepening of the experience. A harmonious and joyous relationship prevails over the spouses’ own needs and they adapt to the world of their beloved.

The loss of closeness and intimacy is described by one of the spouses:

It is like being in a glass bubble; I can’t reach out. It is like not being able to participate …

In this description, there is a pronounced feeling of not taking part. The experience of being cut off, in a glass bubble, expresses helplessness and a loss of life as it was before.

Another spouse expresses a positive experience of being close, how the evening walk became a relaxing break where darkness and silence create harmony:

Every evening, we take a walk around the neighborhood. The same round every night, in all weather; we have a dog and she must go out. This is our most important moment, when we just walk without talking so much. We walk together and go hand in hand in the dark; it is a nice touch. We don’t need so many words; we know each other.

The darkness hides and protects. The silence described is a comfortable silence, a new way to feel closeness and a deeper bond.

Grasping joint opportunities in a new way is described by another spouse:

The most important thing is that we can do things together. You know… now we have the opportunity, later it may be too late.

Togetherness becomes more important and spontaneity provides the everyday life experience of a sense of kinship, still sharing some aspects of life with the loved one. Being together and doing things together is now a priority.

### Conclusive interpretation: lived space, lived time, lived body and lived human relations

The spouses describe in different ways how their lives have changed. Their experience of lived time and lived space becomes different, taking on new shapes. The spouses express the experience of living an imprisoned existence, being hostage in time and space due to their commitment to their loved ones. Obligations, responsibilities, anxiety, fear, and worry keep them trapped at home. The spouses describe how they endure the present, grasping for time to themselves. In their longing for freedom, new strategies and attitudes help the spouses to break free from their imprisonment. The support from others allows them the possibility to create an extended lived space with their partner to be-with-each-other but also a more private lived space by creating room for being-with-myself. Here, the spouses find strength and energy that they bring with them into the everyday being-in-the-world. A new attitude helps them to capture the positive moments in the here and now.

Through the lived body, the spouses retreat both mentally and physically, as they often long for solitude. They try to run away from the experiences of helplessness, loss of intimacy, painful and frustrating feelings, or they try to reach out to others. The lived human relationship is affected by the situation. The spouses adapt to their partners’ being-in-the-world, surrendering their needs and dreams in exchange for a joyous and harmonious everyday life. Support and care from others are essential; being-with-others helps spouses to endure their being-in-the-world. Being-with-myself and being-with-others are necessities, enabling spouses to handle the feelings of alienation from their beloved and the experience of sometimes being perceived as a stranger.

The body’s ambivalence is profound. Through the lived body the spouses cling to their partner’s body as an expression of loneliness, not wanting to let go of what once was. To hold hands is a way to feel closeness, love, mutuality and reciprocity. In the dark, the spouse manages to recreate what once was. Under the cover of darkness, they search for their way back home.

## Discussion

Respondents described their daily lives with their partners as difficult, but they also expressed a deeper existential struggle for their own survival. Heidegger relates the realization of one’s own mortality to the lived time, where the future is not only an opportunity for new experiences but also represents a possible ending in every moment. When a loved one suffers from a serious illness, it creates a sudden awareness that life will end, creating fear and uncertainty about the future. The present and the past are no longer what they were, resulting in feelings of sadness and grief (Svenaeus 2011). The being-in-the-world described by the respondents in this study is no longer homelike; it has become unhomelike not only for the individuals with AD but for their spouses as well. In spite of this, spouses also describe another aspect of their experience that comprises positive moments, the deepening of their lived relationships, and their own internal development and maturity, which gives a more balanced picture of their lifeworld. To cope, they have to surrender and accept their life situation. They exemplify how they reach out to others through the body; for example by touch. Dahlberg et al. (2008) describe how togetherness creates a common space where people share each other’s lifeworld, a constant exchange with the world, in the world.

The spouses in this study also felt that they needed more time and an extended lived space. They expressed feelings of an imprisoned existence that can be related to the unhomelike experience described by Svenaeus (2000). The spouses also expressed a strong wish to take care of their own needs, both through being alone and by being with others, forming supporting relationships. This is confirmed by other studies. Serrano-Aguilar et al. (2006) studied links between the caregiver burden and health problems. They found that caregivers are clearly at risk of experiencing less time for themselves and decreased mobility. Vellone et al. (2008) found that concern for the future may impact caregivers’ life experience and that experiencing peace and quiet is an important factor for their wellbeing. The well-being of the spouse influenced their partners’ well-being and behavior. Their creativity and capacity to create a safe and stimulating environment had a calming effect on their partners.

In this study, spouses described how their partners’ being-in-the world influences their lives and how the lifeworld of the spouses and their partners are tightly intertwined. Öhman (2007) concluded that the expectations and responses of relatives affected the self-image and ability to cope of individuals with dementia. Thomas et al. (2006) found that patients’ well-being and perception of quality of life were strongly associated with caregivers’ quality of life and well-being.

## Conclusion

The present findings may improve our understanding of what it means to live with a partner suffering from early-onset AD, and they could be also be applied to other similar situations. Healthcare professionals, especially in dementia care, can ease the everyday life of spouses by helping them to find strategies to manage feelings of unhomelikeness and guilt and confirm their feelings of discomfort. They can provide an extended lived space, helping spouses to adjust to a new way of life, to regain homelikeness. Here, social assistance and support groups could be valuable resources (SBU 2008; Waite et al. 2004).

Our findings demonstrate the importance of paying attention to spouses’ being-in-the-world as the basis of their existence, because their complex and demanding life situation differs compared to that of spouses of older people with AD and this may affect clinical recommendations.

However, the most important issue that can be raised in relation to findings in our study concerns its implications for professional approach and the meaning of care. The study’s phenomenological point of departure challenges healthcare professionals with the demand to take into consideration the complexity of the lifeworld of patients and spouses. We suggest that caring from this perspective hides something different, something that could be more difficult to face: the existential depth of human life and human vulnerability that touches our innermost. If we choose, as professionals, to encounter the spouses in their role as caregivers, we might unconsciously avoid the existential challenge of their lifeworld. We treat spouses as relatives and caregivers but do we have the courage to face them as fellow human beings?

From a lifeworld perspective, the professional experience is also a personal experience because it involves sharing our common existential conditions of life (Schuster 2013). Encountering the existential vulnerability of spouses implies the courage to see beyond the image of a caregiver, leaving the security of good, practical advice. This has implications to how we understand what care is, with its fundamental obligation to alleviate suffering. Being a caregiver in a lifeworld perspective means to take a step into a reciprocity with the other. It means to acknowledge the other as being an expert on his/her own life. Galvin and Todres (2011) describe this as humanly sensitive care through embodied relational understanding involving the knowing of head, hand and heart.

As professional caregivers we think and we act. When it comes to the heart the challenge is to keep the focus on the other persons experiencing of his/her life situation. It is also important to understand that reciprocity in professional relations does not mean being private with patients and spouses, but it does mean personal involvement. This kind of relation could be described as professional friendship. The difference between professional friendship and private friendship lies in the caregivers’ awareness of his/her ethical responsibility as a fundamental obligation in professional care. This means to receive a vulnerable other, even if this can be painful (Schuster 2006). Supervision as an existential dialogue could be of value in helping professionals to meet these challenges. Healthcare professionals might have to question their previous roles and attitudes, their own being-in-the-world.

### Validation of the study

What emerged from the four existential themes helped us to capture a picture that illuminated spouses’ experiences from a more holistic perspective. However, it is important to keep in mind that the using existentials is only one possible way among others to understand the meaning of a holistic perspective in research.

The validity of this study may be assessed on the basis of the respondents’ descriptions of their life situation at the time the interviews were performed. The authors considered different aspects of validity throughout the entire research process: a communicative validity that imposes a dialogue about the interpretations among other researchers and a pragmatic validation regarding the effect of the findings on practice (Kvale 1989). To further validate the findings, quotations were used to illustrate and provide concrete examples of the thoughts, feelings, or moods of the persons interviewed (Schuster 2013).

The authors problematized their own pre-understanding and discussed and questioned feelings and experiences throughout the research process.

The interpretations in this study are based on how ten spouses describe their experiences of living close to someone with early-onset AD. The connection to a theoretical thinking by means of the existentials helped us to understand the particular persons acting, reflecting and experiencing in the light of more abstract ideas of the meaning of being human. The strength of interpretative studies is that they can give deeper insights and that their meaning can be transferable to other similar life situations.

### Further investigation

It would be of interest to further investigate how the spouses with a partner affected by early-onset AD cope with the unpredictability of the development of the disease in a longitudinal study and to explore the lifeworld of even other family members.
